# Traumatic Retropharyngeal Hematoma in a Patient Taking Clopidogrel

**DOI:** 10.1155/2018/6147473

**Published:** 2018-08-13

**Authors:** David P. Betten, Jennifer L. Jaquint

**Affiliations:** Department of Emergency Medicine, Michigan State University College of Human Medicine, Sparrow Health System Lansing, Michigan, USA

## Abstract

The development of a retropharyngeal hematoma may lead to acute airway compromise requiring emergent airway stabilization. We describe the development of a retropharyngeal hematoma in an elderly woman who sustained a fall from standing approximately 10 hours prior to symptom onset who was taking the antiplatelet agents clopidogrel and aspirin. This delayed onset of rapid airway compromise secondary to a retropharyngeal hematoma following a fall in a patient taking clopidogrel is an unusual and potentially life threatening event.

## 1. Introduction

Retropharyngeal hematomas are an uncommon yet potentially life threatening emergency due the proximity to the upper airway. Frequently reported in the setting of trauma with or without associated bony injury, retropharyngeal hematomas have been described following hemorrhagic transformation of underlying mass lesions and infections, as a complication of central line and nasogastric tube placement, in addition to occurring as a spontaneous unprovoked bleeding event [1–5]. Individuals with hemophilia and those taking anticoagulants such as warfarin appear to be at an increased risk of retropharyngeal hematomas [4, 6, 7]. We report a patient on the antiplatelet agents clopidogrel and aspirin who sustained a mechanical fall from standing with symptoms onset secondary to a large retropharyngeal hematoma identified 10 hours following her injury.

## 2. Case Presentation

**Figure 1:**
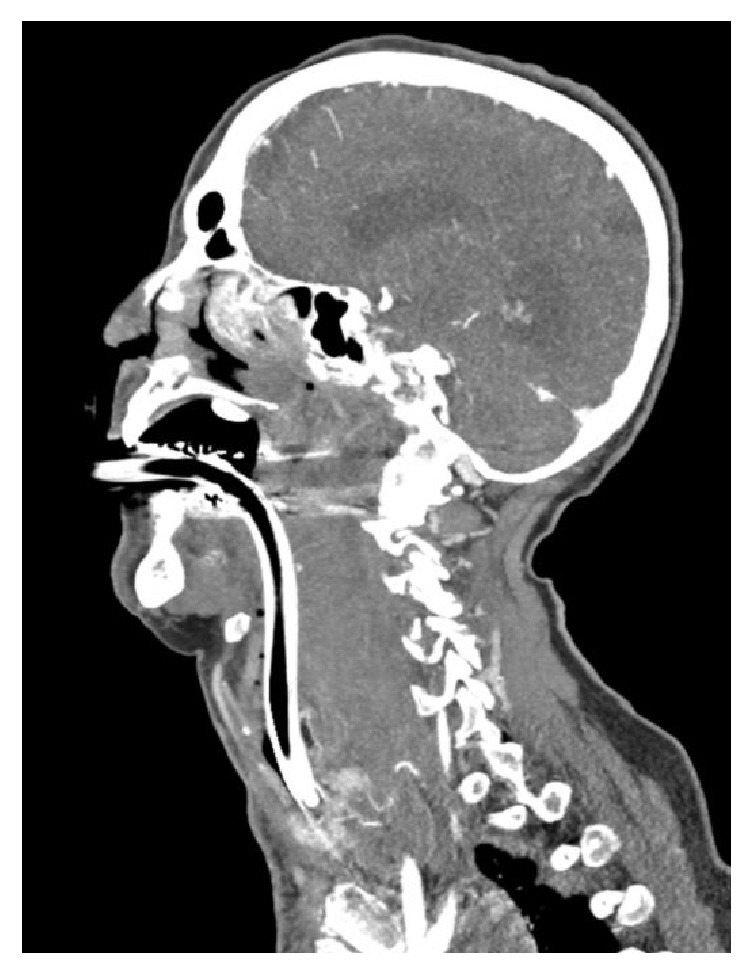
Sagittal plane CT angiogram of neck following intubation demonstrating a large retropharyngeal hematoma and anterior airway displacement.

An 81-year old female with a history of coronary artery disease, hypertension, and thrombocytosis suffered a witnessed trip and fall onto a nightstand. The patient took 75 mg of clopidogrel daily in addition to an 81 mg aspirin tablet. She reported a mild headache however had no change from her baseline mentation per family members with no evidence of obvious injury aside from a small area of ecchymosis near a small forehead laceration. She remained up and ambulatory with no further complaints. Ten hours after her injury the patient presented to the Emergency Department with stridorous and agonal respirations with a profoundly decreased level of consciousness. She was noted to have developed extensive ecchymosis on the anterior portion of her neck and chest. Her symptoms had begun rapidly shortly prior to arrival while lying in bed. Family reported that she had been in the constant company of her husband with no further falls or injuries that had occurred since her fall. The patient was intubated upon hospital arrival due to respiratory extremis with obvious swelling and crepitus noted on neck examination. A noncontrast CT scan of head was unremarkable while there was demonstration of a large retropharyngeal hematoma measuring 3.6 cm by 5.3 cm by 20 cm on a CT of the cervical spine with no evidence of fracture. Her hemoglobin was 9.5 gm/dL and platelets were 1234 per deciliter, with an INR of 3 and a slightly below normal and activated partial thromboplastin time of 23.9 seconds (reference range 25-35 seconds). A CT angiogram of the neck was subsequently obtained demonstrating active bleeding from the anterior ligaments of the vertebral column that was not felt to be amenable to embolization (Figure 1). Given the extent of the hematoma intraoral surgical evacuation was performed with bleeding from the anterior vertebral spine controlled with Bovie cauterization, placement of topical thrombin, and drain placement. No reaccumulation of hematoma was noted during her hospital course. The patient unfortunately expired 12 days from the date of admission from presumed aspiration pneumonia and multisystem organ failure.

## 3. Discussion

The timing of symptom onset for individuals sustaining a retropharyngeal hematoma following a traumatic event is variable with individuals presenting with both sudden onset of symptoms and a more insidious progression. Frequently described signs and symptoms include dyspnea, stridor, dysphagia, and dysphonia with neck pain and swelling often noted [8]. A classic clinical presentation pattern may be found similar to our patient termed “Capps' Triad” that includes ecchymosis over the neck and anterior chest, anterior displacement of the trachea, and evidence of tracheal and esophageal compression [9]. If tolerated, direct visualization of the posterior pharynx may identify a swollen and purple tinted mass suggestive of underlying hematoma. While a history of trauma is typically provided, spontaneous unprovoked retropharyngeal hematomas may occur, typically in those with bleeding diathesis or taking oral anticoagulants [6, 7].

The stabilization of the patient's airway is paramount given the potential for rapid expansion of the hematoma into the loose connective tissue lying between the pharynx and alar layer of the prevertebral fascia. Hematoma formation may be extensive extending from the base of the skull to the tracheal bifurcation [10]. In the setting of impending airway compromise, immediate intubation with either direct laryngoscopy or fiberoptic intubation should be performed with all efforts made to minimize airway trauma given the theoretical concern of hematoma rupture. Emergent tracheostomy can be considered as an alternative yet infrequently utilized option. Plain radiographs will typically identify an enlarged prevertebral space with CT or MRI able to further characterize the extent of hematoma formation.

Several approaches to management of retropharyngeal hematoma have been successfully implemented. Reversal of coagulopathy in those taking anticoagulants should be performed if indicated. Conservative management has been successfully utilized with an understanding that this may entail a period of extended observation of up to three weeks until a complete resolution of the symptoms and underlying hematoma occurs [8].The use of angiography guided embolization or surgical exploration should be considered in the setting of progression of the expanding hematoma and marked airway obstruction [10, 11]. Potential sources of bleeding identifiable on angiogram and surgical exploration are multiple including the anterior longitudinal ligaments, inferior thyroid artery, and the thyrocervical artery [1, 11–13]. Although frequently administered, the benefit of steroids and antibiotics for retropharyngeal hematoma is unclear [4].

Retropharyngeal hematomas have been well described in the setting of minor and major trauma. A delay of symptoms onset is less common and inhibition of platelet function given use of both clopidogrel and aspirin would appear to have placed this patient at increased risk. A similar case was reported by Lazott et al. that involved a patient taking no antiplatelet agents or anticoagulants who sustained a fall with initial CT imaging demonstrating a C1 anterior arch fracture with mild prevertebral soft tissue prominence but no hematoma [13]. The patient 20 hours later developed rapidly progressing respiratory distress requiring intubation with an MRI performed identifying a large retropharyngeal hematoma compressing the airway.

The use of anticoagulant and antiplatelet medications has been clearly associated with an overall higher risk of immediate bleeding episodes and death from intracranial hemorrhage [14, 15]. Delayed CT evidence of intracranial bleeding for patient's taking warfarin following normal imaging studies in addition to numerous case reports of delayed symptom onset with subsequently identified retropharyngeal hematoma in those taking warfarin offers support to a period of close observation for both delayed intracranial and retropharyngeal bleeding episodes in this population [8, 16–18]. For individuals taking clopidogrel, hematoma formation is nealy always noted on initial head CT. Extrapolation of this to pattern to neck and C-spine injuries would suggest rapid onset of bleeding to be evident on initial CT scans of the neck with delayed bleeding unlikely to occur. The role of platelets in the initial phase of clot formation rather than later on in the formation of secondary hemostasis would lend further support towards a great likelihood of immediate bleeding that would require early patient imaging with delayed bleeding seeming less likely.The development of a delayed progression of bleeding in our patient is unique; an initially minimally symptomatic hematoma, which rapidly progressed to be life threatening, could be hypothesized to have occurred. Should additional cases be identified such as this, a management approach that involves close observation and consideration of repeat imaging may be appropriate.

This case is the first to our knowledge of an individual on clopidogrel sustaining an injury resulting in a delayed symptom onset a retropharyngeal hematoma. The use of clopidogrel and aspirin may have contributed to this unfortunate event. This later onset of symptom progression of a life threatening bleeding event is an unusual injury pattern that may occur and one that healthcare providers and patients should be aware of.
